# Fe-Co-B Soft Magnetic Ribbons: Crystallization Process, Microstructure and Coercivity

**DOI:** 10.3390/ma13071639

**Published:** 2020-04-02

**Authors:** Anna Wojcik, Wojciech Maziarz, Maciej Kowalczyk, Robert Chulist, Maciej Szlezynger, Pawel Czaja, Lukasz Hawelek, Przemyslaw Zackiewicz, Patryk Wlodarczyk, Aleksandra Kolano-Burian

**Affiliations:** 1 Institute of Metallurgy and Materials Science, Polish Academy of Sciences, 25 Reymonta Str., 30-059 Krakow, Poland; w.maziarz@imim.pl (W.M.); r.chulist@imim.pl (R.C.); m.szlezynger@imim.pl (M.S.); p.czaja@imim.pl (P.C.); 2 Faculty of Materials Science and Engineering, Warsaw University of Technology, 141 Woloska Str., 02-507 Warsaw, Poland; maciej.kowalczyk@pw.edu.pl; 3 Lukasiewicz Research Network—Institute of Non-Ferrous Metals, 5 Sowinskiego Str., 44-100 Gliwice, Poland; lukaszh@imn.gliwice.pl (L.H.); przemekz@imn.gliwice.pl (P.Z.); patrykw@imn.gliwice.pl (P.W.); aleksandra.kolano-burian@imn.gliwice.pl (A.K.-B.)

**Keywords:** soft magnetic ribbons, in situ transmission electron microscopy, coercivity

## Abstract

In this work, a detailed microstructural investigation of as-melt-spun and heat-treated Fe_67_Co_20_B_13_ ribbons was performed. The as-melt-spun ribbon was predominantly amorphous at room temperature. Subsequent heating demonstrated an amorphous to crystalline α-(Fe,Co) phase transition at 403 °C. In situ transmission electron microscopy observations, carried out at the temperature range of 25–500 °C and with the heating rate of 200 °C/min, showed that the first crystallized nuclei appeared at a temperature close to 370 °C. With a further increase of temperature, the volume of α-(Fe,Co) crystallites considerably increased. Moreover, the results showed that a heating rate of 200 °C/min provides for a fine and homogenous microstructure with the α-(Fe,Co) crystallites size three times smaller than when the ribbon is heated at 20 °C/min. The next step of this research concerned the influence of both the annealing time and temperature on the microstructure and coercivity of the ribbons. It was shown that annealing at 485 °C for a shorter time (2 s) led to materials with homogenous distribution of α-(Fe,Co) crystallites with a mean size of 30 nm dispersed in the residual amorphous matrix. This was reflected in the coercivity (20.5 A/m), which significantly depended on the volume fraction of crystallites, their size, and distribution.

## 1. Introduction

Among several groups of soft magnetic materials, Fe-based amorphous and nanocrystalline alloys are extremely interesting from both a scientific and an application point of view [1,2,3,4,5,6,7,8,9,10,11,12,13,14,15,16,17]. They exhibit not only optimal soft magnetic properties (e.g., low coercivity (H_c_) and high permeability (µ’)) but also are characterized by relatively low magnetic core losses (P_s_) in comparison with other materials. For this reason, they find various applications in motors, transformers, actuators, sensors, and electronic communication devices [3]. However, soft magnetic properties are sensitive to chemical composition as well as microstructural features of materials [18]. It was reported that nanocomposites consisting of α-Fe crystallites with sizes smaller than 35 nm and surrounded by an amorphous matrix showed low coercivity and high permeability [1,18]. This behavior, explained by Herzer [18] in 1989, is based on a random anisotropy model in which the averaging of magnetocrystalline anisotropy plays a key role. Thus, for this reason, α-Fe crystallites have to be smaller than the natural exchange length, which equals 35 nm for Fe. Customarily, nanomaterials are fabricated in a multistage process, which typically is realized in two steps: (1) production of amorphous ribbons by rapid quenching techniques and (2) crystallization of α-Fe nanocrystals involved by heat treatment [1,19]. The most important conditions, which allow for obtaining a fine and homogenous microstructure, are (i) large nucleation rates and (ii) small crystallite growth rates. Moreover, the crystallization process is also dependent on chemical composition. For systems containing Cu, heterogeneous nucleation of the α-Fe phase on Cu-clusters occurs, while the addition of other elements inhibits the crystallite’s growth [20]. In Cu-free alloys, homogenous nucleation takes place, while nucleation density is very sensitive to annealing temperature and heating rate [21].

Despite the work that has been done on Fe-Co-B ribbons, [22,23] including both structural and magnetic properties in the function of chemical composition and heat treatment conditions, there are still not that many detailed studies on the complex microstructural investigation of Fe-Co-B in the context of ultrarapid annealing (URA). Recently authors presented the analysis of the Fe_80−x_Co_20_B_x_ (x = 12–15 at.%) ribbons and discussed the effect of the chemical composition (boron content) and subsequent conventional heat treatment conditions on the structure, microstructure, and magnetic properties of these materials. It was shown that with the increase of boron content, the crystal structure of ribbons changes as follows: fully crystalline (12 at.% of B) → amorphous +crystalline (13 at.% of B) → fully amorphous (14 and 15 at.% of B). Moreover, magnetic properties such as the saturation magnetic flux density (B_s_) value as well as coercivity were examined. The highest value of B_s_ was found to be for both as-spun and annealed Fe_67_Co_20_B_13_ ribbons, while Fe_67_Co_19_B_14_ ribbon exhibited the narrowest coercivity [22]. Thus, based on the aforementioned reports, we selected the optimal composition of Fe_67_Co_20_B_13_ to be promising for further investigation.

In this work, an attempt is made to study the crystallization process, structure, microstructure, and magnetic properties of as-spun and annealed Fe_67_Co_20_B_13_ ribbons. The in situ TEM observations during heating with two heating rates of 20 and 200 °C/min were carried out with the aim to understand the evolution of the crystallization process in both heating conditions. High-energy synchrotron measurements allowed one to estimate the amount of crystalline phase with respect to the amorphous ones. Microstructural observations, especially using high-resolution TEM, gave substantial information about the size and distribution of crystallites in an amorphous matrix as well as the microstructural details on an atomic scale. Furthermore, the correlation of structural–microstructural features and the effect on the crystallization process with magnetic properties was also discussed.

## 2. Materials and Methods

Fe_67_Co_20_B_13_ master alloy was prepared by induction melting from chemical elements Fe (99.85%), Co (99.9%), and binary compound FeB_18_ in an argon atmosphere. Then, the as-cast ingot was induction melted in an argon atmosphere and ejected with 26 kPa overpressure onto a copper wheel rotating with a linear speed of 34 m/s. The obtained ribbon (termed Ribbon 0) had 10 mm in width and 16 µm in thickness. Subsequent annealing at three temperatures for various times was performed at (i) 370 °C/60 s, (ii) 410 °C/30 s, and (iii) 485 °C/2 s, in a specially designed block heating system for the ultrarapid annealing technique, hereafter referred to as Ribbon 1, Ribbon 2, and Ribbon 3, respectively. The main parts of the heating system are two bulky copper blocks, heated up to the appropriate temperature. The temperature is stabilized by the PID controller, in terms of the thermocouple’s signal, situated in one of the blocks close to the sample’s surface. The microstructure of ribbons was examined using a Tecnai G2 Transmission Electron Microscope (TEM) (FEI, Eindhoven, The Netherlands) equipped with an energy-dispersive X-ray microanalyzer (EDX) (EDAX, Mahwah, NJ, USA) and a High Angle Annular Dark Field (HAADF) detector (Fischione, Pennsylvania, Pittsburgh, PA, USA). The Gatan 628 heating holder was used for the in situ experiments. Thin foils for TEM observations were prepared with TenuPol-5 double jet electropolished using an electrolyte of perchloric acid (20 vol.%) and methanol (80 vol.%) at a temperature of about −20 °C. Room temperature high-energy, wide-angle X-ray diffraction measurements were carried out at DESY synchrotron in Germany, Hamburg using the beamline P07 (87.1 keV, λ = 0.0142342 nm). The samples were rotated 180° around the ω axis in order to obtain diffraction with good grain statistics [24]. The amount of crystalline phase was calculated using Origin 2018 Academic software, from the integrated area of each peak using the following equation: area of crystalline peaks/(area of crystalline peaks+amorphous peaks). The background correction was performed using HighScore Plus software. Thermoanalysis was performed by a differential scanning calorimetry (DSC) using a thermal analyzer (Netzsch DSC 404C Pegasus, Netzsch-Gerätebau GmbH, Selb, Germany)) instrument with a heating rate of 20 °C/min. Magnetic measurements were performed in a hysteresis loop tracer, specially designed for soft magnetic materials in the ribbon shape attachment. Properties of extremely soft magnetic materials were measured with the high sensitivity in the magnetic field with a range of ±660 A/m. The measuring unit was based on the idea described by Kulik et al. [25].

## 3. Results and Discussion

### 3.1. Characterization of the as Spun Fe_67_Co_20_B_13_ Ribbon

**Figure 1:**
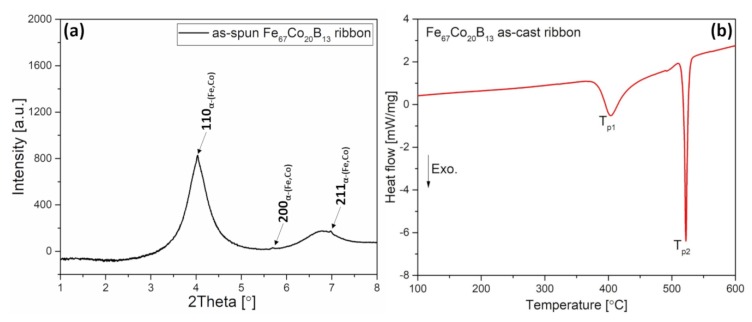
(**a**) High-energy X-ray diffraction pattern and (**b**) differential scanning calorimetry curve recorded during heating for as-spun Fe_67_Co_20_B_13_ ribbon.

**Figure 2:**
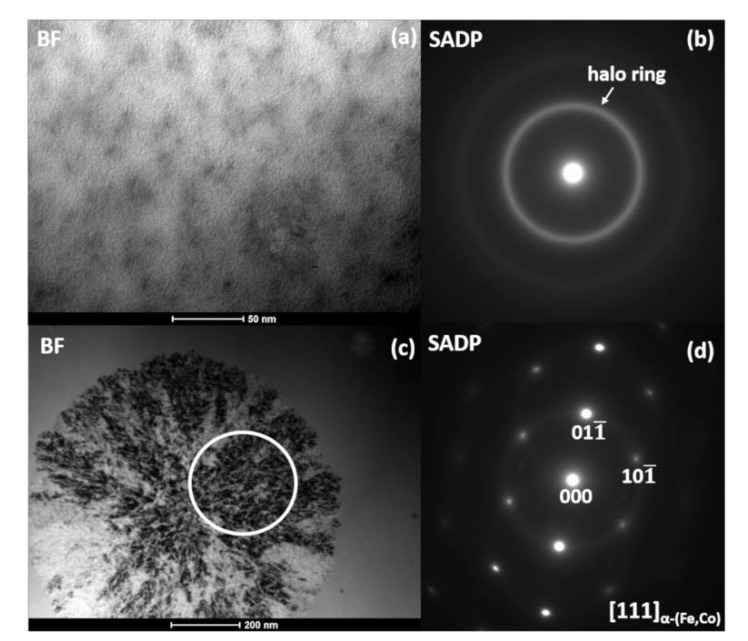
Bright-field (BF) images (**a**,**c**) and selected area diffraction patterns (SADPs) (**b**,**d**) for as-spun Fe_67_Co_20_B_13_ ribbon.

Figure 1a shows the integrated high-energy synchrotron radiation diffraction patterns for the as-spun ribbon. According to the synchrotron diffraction patterns, the as-spun ribbon is almost fully amorphous with a characteristic broad diffused diffraction halo. Only a trace amount of crystallites (about 2%) in the amorphous matrix is detected. Very low peaks are observed at the 2Theta angles (~4.0, 5.6, and 7.0 degrees) that indicate the presence of a negligible amount in the primary α-(Fe,Co) phase. Han et al. investigated the influence of boron addition on the structure of ribbons and showed the two-phase structure of Fe_67_Co_20_B_13_ as-spun ribbon [22]. The differential scanning calorimetry (DSC) curve (Figure 2b) recorded on heating for Fe_67_Co_20_B_13_ as-spun ribbon showed the two-stage crystallization process. The first exothermic peak (T_p1_), with the minimum at 403 °C, corresponds to the crystallization of the α-(Fe,Co) phase. The second peak (T_p2_) occurring at 522 °C is related to the crystallization processes of iron and/or cobalt borides. Based on DSC curves, the temperatures for the heat treatment process have been selected, i.e., below and above the T_p1_ value.

**Figure 3:**
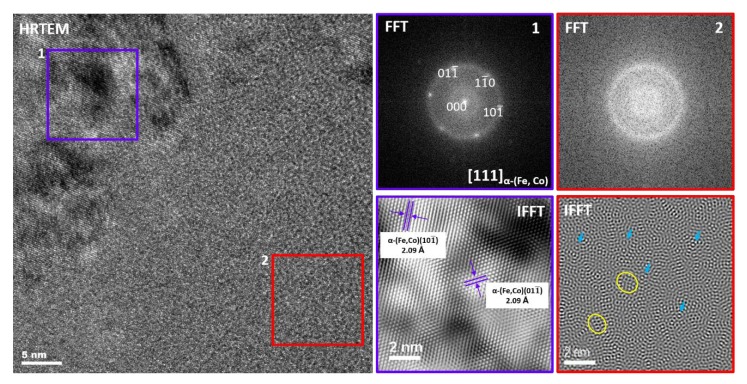
High-resolution transmission electron microscopy (HRTEM) image and fast Fourier transform (FFT), and an inverse fast Fourier transform (IFFT) taken from crystalline (1) as well as amorphous (2) regions of as-spun Fe_67_Co_20_B_13_ ribbon.

Figure 2 demonstrates bright-field (BF) images and the corresponding selected area electron diffraction patterns (SADPs) taken from different areas of the as-spun Fe_67_Co_20_B_13_ ribbon. Besides the amorphous phase (Figure 2a,b) with a characteristic amorphous halo ring (marked by white arrow), preliminary nanocrystalline regions have been found (Figure 2c,d). The SADP has been indexed in accordance with the bcc α-(Fe,Co) crystal structure. The size of crystalline regions was estimated to be between 100 and 200 nm. Moreover, the high-resolution transmission electron microscopy (HRTEM) micrograph, fast Fourier transform (FFT), and inverse fast Fourier transform (IFFT) images taken from (1) crystalline and (2) amorphous regions, marked with squares, are presented in Figure 3. The FFT obtained from the HRTEM image pointed by square 1 can be well indexed based on the α-(Fe,Co) structure confirming SADPs results. Additionally, the lattice fringes of the observed crystallite correspond to the (110) planes of the *bcc* α-(Fe,Co) phase. The area of square 2 reveals the existence of local atomic ordered regions with a size of about 1–2 nm, called “nanocrystalline (atomic) clusters” randomly dispersed in the amorphous matrix (highlighted by yellow ovals). Furthermore, onion-like contrasts are marked by blue arrows. This kind of local microstructure was already reported and described in Fe-Si-B-P-Cu systems [19].

### 3.2. In Situ TEM Heating Observations

**Figure 4:**
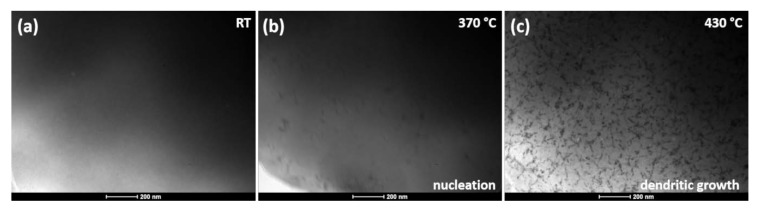
Set of BF microstructures recorded for the samples in-situ heated at 200 °C/min.

The in situ TEM experiments were performed with two heating rates of 20 and 200 °C/min to compare and recreate conditions typically employed during the experimental heat treatment. During the in situ TEM experiments, carried out with a heating rate of 200 °C/min, the evolution of microstructure was observed (Figure 4a). At 375 °C, in consistence with the DSC scan, the crystallization nuclei were revealed, followed by the dendritic growth of crystals (430 °C). The same phenomenon was reported in [23] for Fe_85-x_Co_x_B_15_ alloys for x within the range of 12 < x < 25 at.%. The presence of the crystallized nuclei at this low temperature was associated with a phase separation taking place within the amorphous matrix. This led to the formation of a dendritic α-(Fe,Co) structure, whereas the majority of the sample volume remained in its amorphous state.

**Figure 5:**
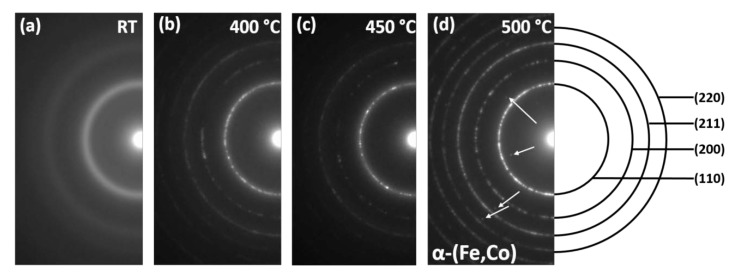
Electron diffractions patterns obtained at different temperatures for samples heated in situ with a heating rate of 200 °C/min.

More significant differences were observed in the electron diffraction patterns (SADP) with the increase of temperature (Figure 5). The SADP taken at room temperature showed two typical diffused halo rings corresponding to the most intense reflections of the α phase, i.e., (111) and (211). At 400 and 450 °C, diffused spots appeared on the first ring (counting from the center of the diffraction pattern) and also on an additional diffraction ring that appeared between the first and the second halo ring. At 500 °C, all the diffraction rings are well-developed, and they correspond with high accuracy to the crystallographic planes of the α-(Fe,Co) phase. In addition, one can see individual reflections marked by arrows, which are characteristic of the Fe_2_B phase. In order to compare the microstructural changes with respect to the heating rate applied (20 and 200 °C/min), the BF image analysis was supplemented with measurements of crystallite sizes (dendrite arms cross-sections) at various temperatures.

**Figure 6:**
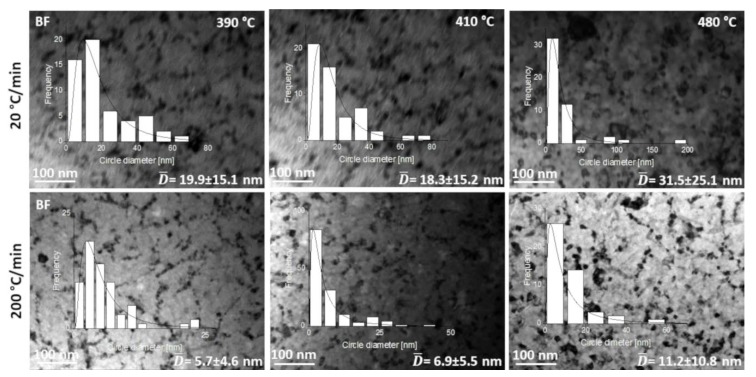
Set of BF microstructures recorded at different temperatures during in situ heating at 20 and 200 °C/min and the corresponding histograms of crystallite size distribution.

Figure 6 shows a set of microstructures in the BF mode recorded for different temperatures during in situ heating at 20 and 200 °C/min. Histograms of crystallite size distribution and their average values are attached to individual images of microstructures. The histograms have been fitted by a lognormal function similarly, like in the case of rapid annealed Fe-Si-Nb-B-Cu soft magnetic amorphous alloys [13]. From Figure 6, one can see that in both cases, the same crystallization mechanism occurs, including nucleation and dendritic growth. However, the crystallite size for samples heated at 20 °C/min is three times larger than the crystal size of samples heated at 200 °C/min for the same measurement temperatures. In addition, by analyzing the nature of the histograms, it can be concluded that in the first case (20 °C/min), a bimodal crystallite size distribution occurs at all temperatures. In contrast, for the second case at temperatures of 410 °C and 480 °C, unimodal normal type distribution occurs. In summary, it can be stated that the crystallization process of amorphous Fe_67_Co_20_B_13_ ribbons at 20 and 200 °C/min heating rates carried out by in situ TEM leads to visibly different microstructures in respect of the particular size and distribution of the crystalline phase, which in turn leads to various magnetic properties.

**Figure 7:**
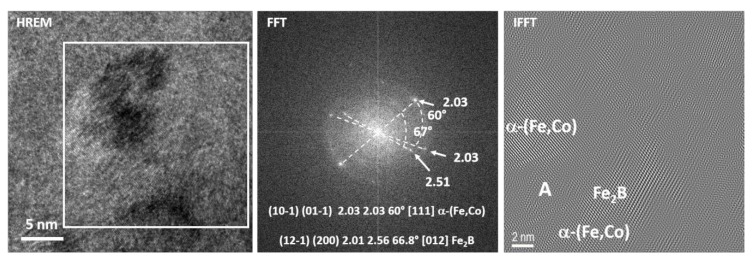
HREM, FFT, and IFFT images of the sample in situ heated up to a temperature of 500 °C with a heating rate of 200 °C/min.

Figure 7 shows the HREM image together with the corresponding FFT and IFFT images of the sample heated in situ to a temperature of 500 °C with a heating rate of 200 °C/min. Reflections corresponding to interplanar distances such as 2.51 and 2.03 Å, as well as diffusion rings corresponding to the amorphous phase, are well visible on the FFT image taken from the marked area (white square). According to the angle’s measurement, the presence of α-(Fe,Co) and (Fe,Co)_2_B phases with the [111] and [012] zone axes may be confirmed, respectively. Accordingly, the IFFT’s two nano regions corresponding to the α-(Fe,Co) and Fe_2_B phases separated by the amorphous phase can be distinguished (Figure 7 right image).

### 3.3. Characterization of Heat Treated Fe_67_co_20_B_13_ Ribbons

**Figure 8:**
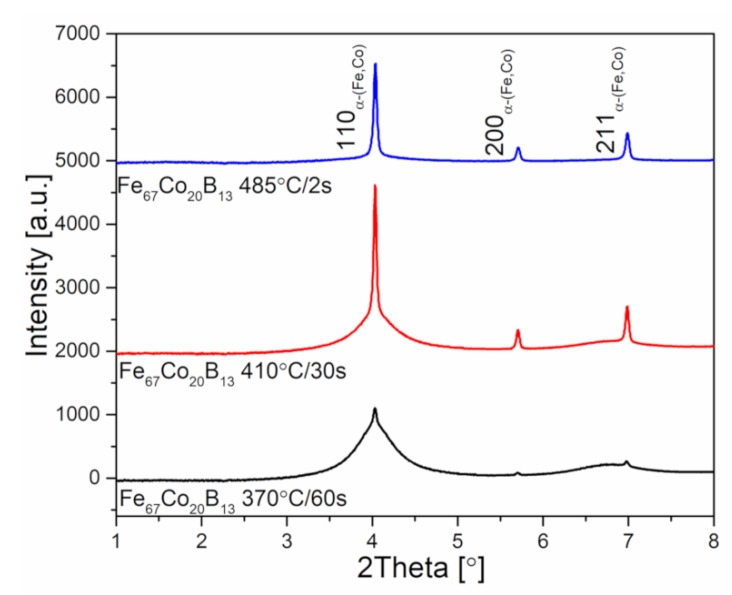
High-energy X-ray diffraction patterns of heat treated Fe_67_Co_20_B_13_ ribbons.

Figure 8 presents the synchrotron X-ray diffraction patterns of heat-treated ribbons. Samples after annealing show a two-phase structure consisting of both amorphous and α-(Fe,Co) crystalline phases. Moreover, the amount of crystalline α-(Fe,Co) phase calculated from X-ray diffraction patterns is found to be 6.5%, 38.7%, and 91.6% for ribbons annealed at 370, 410, and 485 °C, respectively. It is therefore clear that the higher the heat treatment temperature is, the greater the crystalline to amorphous phase ratio.

**Figure 9:**
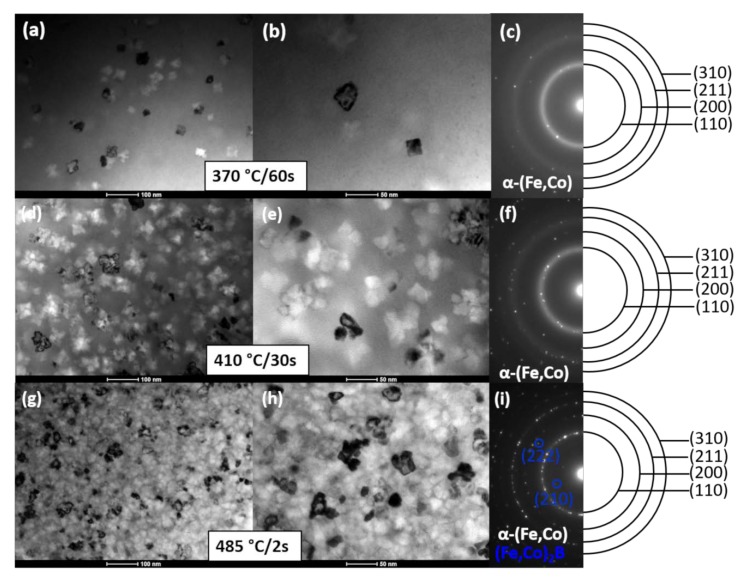
Set of BF images and corresponding SADPs for Fe_67_Co_20_B_13_ ribbons heat-treated under various conditions: 370 °C/60 s (**a**), (**b**), (**c**), 410 °C/30 s (**d**), I, (**f**), and 485 °C/2 s (**g**), (**h**), (**i**).

Figure 9 shows the set of BF micrographs (a, c, e) and SADPs (b, d, f) for Fe_67_Co_20_B_13_ ribbons after heat treatment performed under various conditions (370 °C/60 s, 410 °C/30 s, and 485 °C/2 s). Ribbon 1 has α-(Fe,Co) crystallites with an average crystal size of 53 ± 13 nm, randomly placed in the amorphous matrix. Both the BF image and SADP indicate that the content of the crystalline phase is much smaller than the amorphous phase. Ribbon 2 contains much more α-(Fe,Co) crystal grains compared to Ribbon 1, while the crystallites’ size decreases to 37 ± 8 nm. Ribbon 3 annealed at the highest temperature, within the investigated temperature range at almost fully crystalline, with the small amorphous regions surrounding α-(Fe,Co) crystallites with an average size of 30 ± 8 nm. By the increase of annealing temperature, extra spots appear. The new phase was identified to be iron and/or cobalt borides (Fe,Co)_2_B. The distinction between both phases cannot be made due to the same crystallites structure (I4/mcm) and similar lattice parameters (for Fe_2_B and for Co_2_B). In this case, the amount of borides was almost negligible. However, it is well known that the presence of borides in greater amounts has a negative impact on soft magnetic behavior leading, e.g., to the increase of coercive fields.

**Figure 10:**
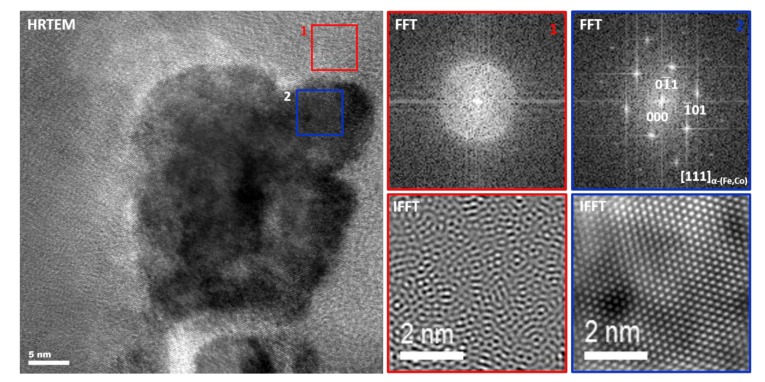
HRTEM image, FFT, and IFFT images taken from the (1) amorphous region and (2) α-(Fe,Co) crystallite for Fe_67_Co_20_B_13_ heat-treated at 485 °C/2 s.

HRTEM micrograph for Ribbon 3 is shown in Figure 10. It proves that α-(Fe,Co) crystallites with a mean size of 30 nm are spread in the amorphous matrix. The aforementioned ribbons, due to their unique microstructure, are called “nanocomposite materials”, where the α-Fe crystallites are embedded in an amorphous matrix, which is beneficial from a magnetic properties point of view.

### 3.4. Magnetic Properties

**Figure 11:**
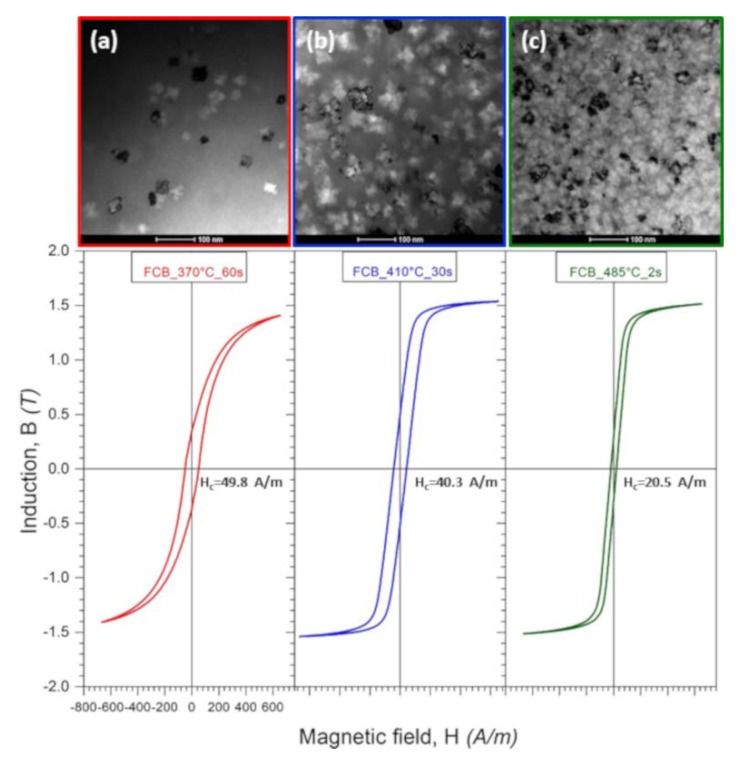
Microstructure (BF images) and B-H curves for Fe_67_Co_20_B_13_ ribbons heat-treated at 370 °C/60 s (**a**), 410 °C/30 s (**b**), and 485 °C/2 s (**c**), show the change of hysteresis loops with the microstructural evolution of ribbons.

Figure 11 presents B-H (where B is induction and H is a magnetic field) loops of heat-treated Fe_67_Co_20_B_13_ ribbons in the correlation with microstructural features apparent in BF images. The coercivity (H_c_) considerably diminishes with the increase of heat treatment temperature from 49.8 A/m for Ribbon 1 to 20.5 A/m for Ribbon 3. This behavior is a consequence of microstructure evolution, including both α-(Fe,Co) crystallite sizes and their volume fraction as well as the distribution in the amorphous matrix. In the case of the ribbon annealed at a temperature of 370 °C for 60 s, slightly below the onset of α-(Fe,Co) phase crystallization peak (Figure 1b), the crystallites are the largest (53 nm), unevenly embedded in the matrix. Here, the α-(Fe,Co) crystallites grow from Fe-rich regions (well observed in HRTEM—Figure 4) while the annealing time (60 s) is sufficient for crystal growth. As a consequence of the existence of larger, heterogeneously distributed α-(Fe,Co) crystallites, the coercive field value is the largest. However, Ribbon 3 annealed at 485 °C for two s is characterized by a fine microstructure, where the crystallites with an average crystallite size of 30 nm are uniformly distributed in the amorphous matrix. In this case, α-(Fe,Co) crystallites are connected with both preexisting nuclei as well as newly formed ones. Short annealing times and a large number of nuclei inhibit crystallites growth. Thus, a low coercive field (20.5 A/m) in this alloy can be explained by the fact that the exchange correlation length is larger than the crystallite’s size. Additionally, it can be noticed that the saturation magnetic induction (B_s_) was estimated to increase subtly with the annealing temperature.

## 4. Conclusions

Based on the in situ TEM experiments and the nano- and microstructure observations, the following conclusions can be drawn: (i) independently of the heating rate, the crystallization process of amorphous Fe_67_Co_20_B_13_ melt-spun ribbons is realized by the nucleation and dendritic growth of α-(FeCo) phase, while the first crystallization effects are manifested at a temperature close to 370 °C; (ii) finer and more homogeneous microstructures are observed in the case of sample heated with the heating rate of 200 °C/min than in the one heated with 20 °C/min; (iii) formation of Fe_2_B phase at 500 °C during heating with the 200 °C/min heating rate is confirmed by the HREM investigations. The aforementioned results prelude the ultrarapid annealing process for Fe-based soft magnetic ribbons being interesting and prospective from the scientific and application point of view. Moreover, we have examined the microstructure of Fe_67_Co_20_B_13_ ribbons after heat treatment performed under various conditions: (1) 370 °C/60 s, (2) 410 °C/30 s, and (3) 485 °C/2 s. These results were then correlated with the coercivity values. It was found that the annealing at the higher temperature (485 °C) for a very short time (2 s) provides a fine, homogenous microstructure resulting in lower coercivity H_c_ = 20.5 A/m and magnetic induction of B > 1.5 T. Further tests, including structural and magnetic studies, are needed to optimize the ultrarapid annealing process of this material.
